# Frequency of micronucleus in oral epithelial cells after exposure to mate-tea in healthy humans

**DOI:** 10.4317/medoral.19570

**Published:** 2014-03-08

**Authors:** Marcelo C. Bortoluzzi, Eduardo B. Campagnoli, José R. Milan, Angélica Reinheimer, Maicon Masson, Diogo L. Capella

**Affiliations:** 1 PhD, Oral Medicine Professor, School of Dentistry, Dentistry Department, Ponta Grossa State University (UEPG) Brazil and Oral Medicine Professor at Oeste de Santa Catarina University (UNOESC), Brazil; 2 PhD, Oral Medicine Professor, School of Dentistry, Dentistry Department, Ponta Grossa State University (UEPG), Brazil; 3 Oeste de Santa Catarina University (UNOESC), Brazil; 4 DDS, Professor at School of Dentistry, Oeste de Santa Catarina University (UNOESC), Brazil

## Abstract

Objectives: The aim of this study was to evaluate the possibility of technique simplification for cytology slides in order to evaluate the frequency of micronuclei (FMic) and conduct a experiment looking to know the FMic of oral epithelial cells of healthy volunteers exposed to mate tea (Ilex paraguarariensis). Material and Methods: This is a laboratorial and nonrandomized trial (quasi-experiment), where the nonusers subjects were exposed to mate-tea, consumed in the traditional way, two drinks, two times a day for a single week. Two cytology of exfoliated epithelial cells were obtained before and after the mate tea exposition. Results: The sample was composed by 10 volunteers. The age ranged from 18 to 33 years (Mean 23; SD5.5). The use of mate tea did not showed significant variation in the FMic (Wilcoxon Signed Ranks Test p= .24). Conclusions: The proposed technique simplification showed to be reliable, without losses when compared to the conventional technique and with the advantage of eliminate toxic substances, becoming simple and practical tool for research in dentistry. The acute exposure to mate tea did not induce an increase of FMic in exfoliated buccal cells of healthy nondrinkers and nonsmokers subjects and may not have genotoxic effect. More human studies are needed before a conclusion can be made on the oral carcinogenic risk of mate tea to humans.

** Key words:**Micronucleus, Oral Cancer, Cytology, Mate tea, Ilex paraguariensis.

## Introduction

The buccal mucosa epithelial cells are an easily accessible tissue for sampling cells through a minimally invasive technique. These cells can also be used to monitor early genotoxic events as a result of potential carcinogens entering the body through ingestion or inhalation. The buccal cell micronucleus (MN) assay has been considered as a useful biomarker of genetic damage caused by environmental pollutants, life-style habits and even inherited genetic defects in DNA repair. MN originates from aberrant mitosis and it has been defined as a microscopically visible round or oval cytoplasmic chromatin mass next to de nucleus. MN consists of acentric chromosomes, chromatid fragments or whole chromosomes that have failed to be incorporated in the daughter nuclei during mitosis. The formation of MN is therefore induced by substances that cause breakage of chromosomes (clastogens) as well as by agents which affect the spindle apparatus (aneugens). Moreover, clinical studies show that the determination of the frequency of micronuclei (FMic) in different cytological preparations can be reproducible (1-16).

Mate tea (or Yerba-Maté, Jesuit’s tea, Chimarrao, or Paraguayan tea) is a tea-like infusion of the herb *Ilex paraguariensis* (IP) largely consumed in the southern Brazilian states as well as Uruguay and Argentina. Mate tea drinkers have been considered to carry high risks for upper aerodigestive tract cancers, but it is conceivable that this high risk may be attributable to confounding by smoking, alcohol, and other exposures, however, it has been demonstrated that IP could reduce the inflammatory cell influx of macrophages and neutrophils and reduced acute lung inflammation in mice exposed to cigarette smoke (17). The anti-inflammatory action of the Mate tea may be related with the decrease in inflammatory cytokine expression, cell influx and cellular metabolic activity and also with promotion of cell survival due to its prevention, interception and repair protection against peroxynitrite, which causes protein nitration, lipid peroxidation, DNA damage and cell death (17,18). Filip *et al.* (2007) (19) sug-gested that the IP extracts could be useful in prevention of oral pathologies since it has natural antioxidants which may have a potential chemoprotective action in oral tissues due to its action of promoting an increase of activity of secreted peroxidase. Peroxidase is one of the most important scavenger enzymes of the antioxidant system of the submandibular glands, acting preventing attack of free radicals and protecting oral mucosa from cellular lysis induced by H2O2 and hydroxyl radicals. Bortoluzzi *et al.* (2011) (20) also observed lower pain intensity scores after third molar surgeries in subjects regularly taking mate tea. Besides this described protective effects of regular mate tea drink, it has been implicated in the oral cancer development (21).

The aim of this study was to propose a technique simplification for cytology MN essay and conduct an experiment to verify the FMic in healthy and nonusers individuals exposed to mate tea.

## Material and Methods

This is a laboratorial based study complemented by a quasi-experimental clinical trial.

-Subjects 

The research procedures utilized in this study were approved by the university ethical committee. All volunteers where verbally and written informed about the risks and the main objectives of this research and participants signed an informed consent form. This experiment was applied to only healthy and young subjects and were excluded of the clinical trial any individual who was taking any medicine, smokers, person who are using any mouthwash, or have received any recent dental treatment or have taken facial or oral radiographs and have clinically healthy oral mucosa and teeth. To the volunteers were asked to do not drink alcohol beverages at least one week before the first buccal smear of exfoliated cells up to the end of the experiment. Were also excluded from this research individuals who declared a regular weekly alcohol beverage consumption, or those who worked with known carcinogens like as gas station attendants, painters, or activities which regularly expose the workers to pesticides or wood dust. Subjects received the same commercially available pack with the mate tea powder as well as the instructions to how to use it. This hot beverage was consumed in traditional way (hot tea) during one week, twice a day and with two drinks for each session (four drinks a day for seven days). After an additional period of seven to nine days the smear of epithelial cells were collected.

-Exfoliated Buccal Cells Sample Collection and Proposed Slide Preparation 

The patient was examined in the dental clinic under dental potent light to assure the healthy conditions of the oral tissues. The oral mucosa scraping to collect exfoliated cells followed these steps: (a) a light mouthwash with tap water was used to reduce debris; (b) the interior surfaces of the right and left cheeks were gently scraped with a wooden, water-soaked spatula; (c) the sample was applied over two glass slides and air dried using the compressed air from the dental clinic, during approximately 1 minute, taking care to do not over dehydrate the sample.

The staining protocol were prepared immediately after the smear collection and followed these steps: (a) five to six drops of giemsa stock solution (Cinética®) was applied directly over the slide for 2 minutes.; (b) the slides were washed in a container with tap water (container 1: 3 to 4 washes, container 2: 2 to 3 washes); (c) the slide was rapidly differentiate in a third container (1,200 ml of tap water and 1 drop of glacial acetic acid (Vetec Quimica Fina®); (d) The slide was then air dried using the compressed air from the dental clinic, during approximately 1 minute, taking care to do not over dehydrate the sample and mounted (Entel-lan®).

-Scoring

The scoring and selection criteria form MN counting followed the rules already described (4,8,13,22). Cells were checked under light microscope with 100x objective magnification and a single researcher made all the counts to avoid interobserver variability. 1000 cells were count and checked for MN before and after mate tea exposure.

The criteria was originally developed by Tolbert *et al.* (22) and they consist of the following parameters for cell inclusion in the cells to be scored: (a) intact cytoplasm and relatively flat cell position on the slide; (b) little or no overlap with adjacent cells; (c) little or no debris; and (d) nucleus normal and intact, nuclear perimeter smooth and distinct. The suggested criteria for identifying MN are: (a) rounded smooth perimeter suggestive of a membrane; (b) less than a third the diameter of the associated nucleus, but large enough to discern shape and color; (c) staining intensity similar to that of the nucleus; (d) texture similar to that of nucleus; (e) same focal plane as nucleus; and (f) absence of overlap with, or bridge to, the nucleus.

-Statistical analysis

Data were tested for normality by the Lilliefors test and pre and post FMic were compared through Wilcoxon (signed-rank test). All statistical analyses were performed using statistical software (BioEstat®, version 5.0; Belém/Pará- Brazil). Differences were considered as statistically significant with p ≤ 0.05.

## Results

**Figure 1:**
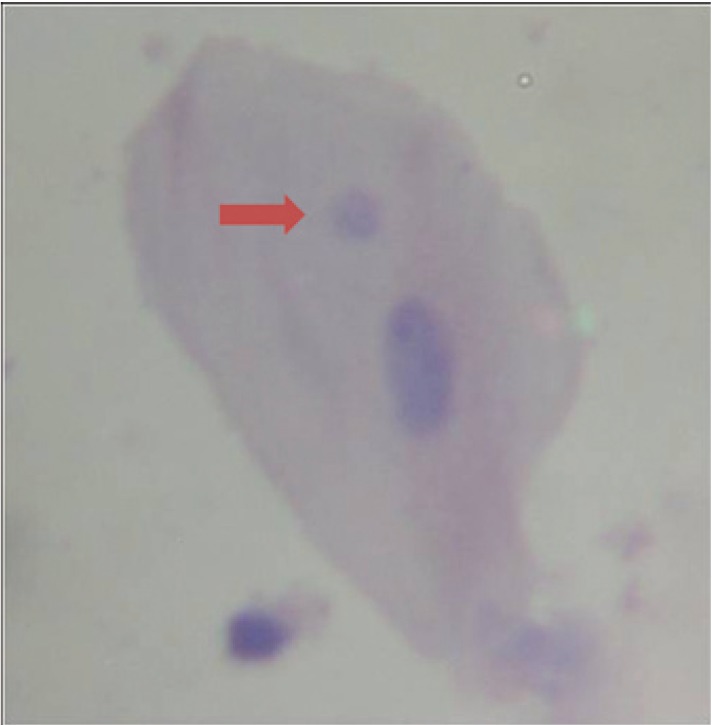
Photomicrography of exfoliated buccal cell showing the presence of micronucleus (arrow) (Giemsa, 100X).

The proposed simplification method for giemsa stain MN slides essay preparation showed to be reproducible, reliable, easy to perform taking less than 10 minutes to be prepared and with a very low cost to be used as a research tool in dentistry (Fig. 1). Moreover, the greatest advantage to use this simple technique is the possibility to avoid the use of toxic chemicals like as methanol and xylene/xylol usually present in conventional techniques ( Table 1). The disadvantage observed in this method proposal was the reduced period for read the slides, which is about 2.5 months and after this period some degree of sample degradation was observed.

**Table 1:**
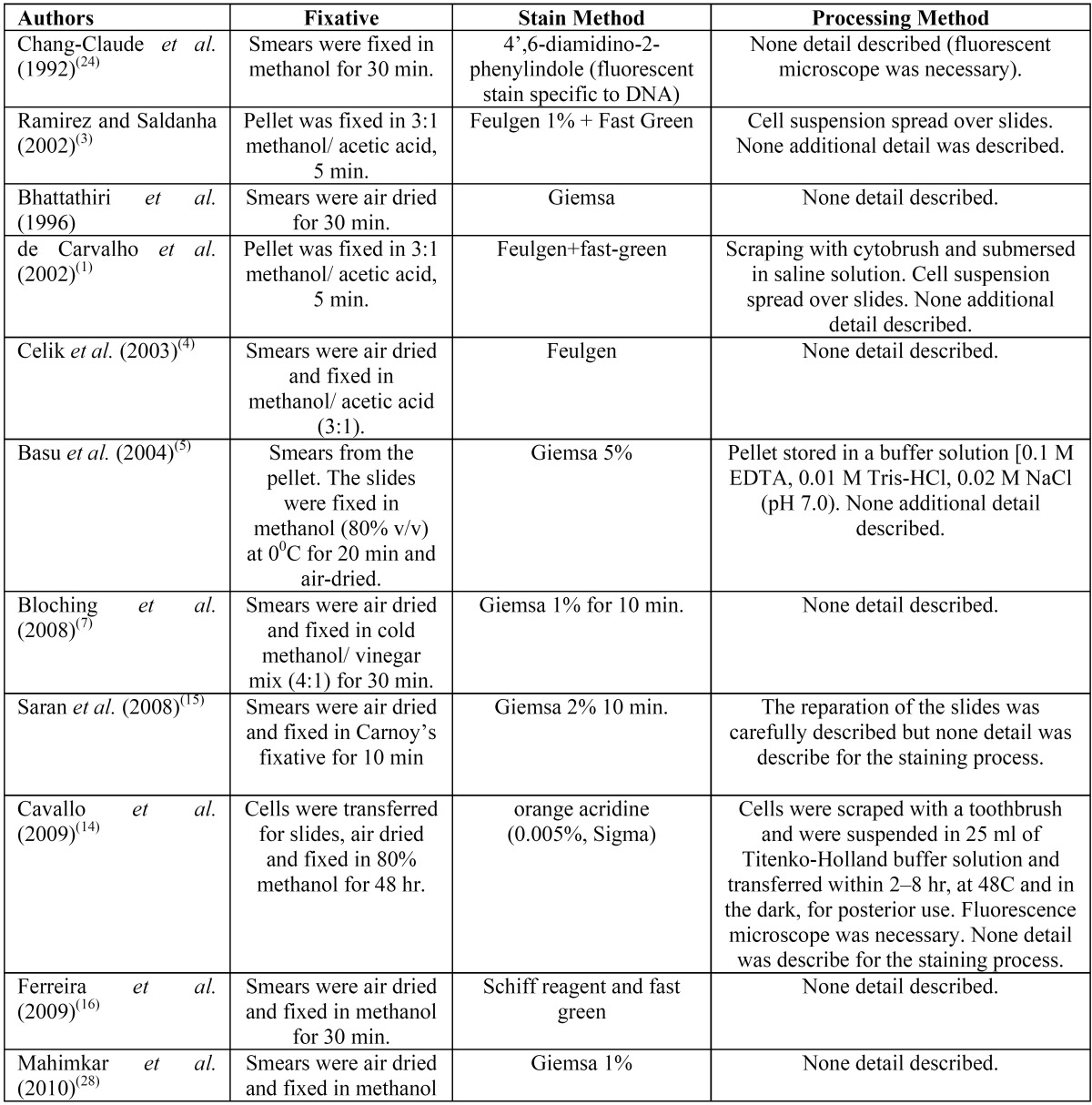
Variability of Micronucleus essay methods including fixative, stain and laboratory processing.

Twenty thousand cells were checked and counted from a sample composed by 10 patients (7 of them were female). The age ranged from 18 to 33 years (Mean 23, SD 5.5). The first statistical analysis did not observe any correlation between age and FMic for before and after MN essay.

The use of mate tea did not showed any relevant FMic changes before intervention (mean 2.7 MN, SD 2.5) and after intervention (mean 2.1, SD 2.2), with no statistical significant differences observed before and after mate tea exposure (Wilcoxon Signed Ranks test, *p*= 0.24).

## Discussion

The study of DNA damage in exfoliated cells collected from the oral cavity holds great promise as a minimally invasive method for monitoring exposition to genotoxic agents and according to Thomas *et al.* (2009) (8) as the buccal cells turn over every 7 to 21 days, it is theoretically possible to observe the genotoxic effects of an acute exposure after this period. Generally, all revised literature for this study confirms that the MN essay from exfoliate buccal cells is an useful biomarker to evaluate the genotoxic effect and consequently measure the damage to the human DNA from a diverse source, however, other studies have questioned its ability to predict or indicate future oral cancer development (23,24).

Besides this manuscript did not attempt to compare different techniques we have done both conventional and simplified technique for giemsa stain just to check the stain pattern. It was observed that the simplified technique was equivalent or better than the conventional due to basically less washing steps, none alcohol to dehydrate and hydrate steps and none xylene use. For this research protocol development several issues was taken into account, considering ethical reasons to acutely expose patients to use mate tea, the literature, the technique suitability and simplicity, costs and mainly the reagents safety which would be suitable for use directly in dentistry clinic as a research tool. The evaluation of the literature shows that a variety of different stains is used in MN studies and the most specific stains are those called “DNA-specific”, and among those could be named Feulgen, acridine orange, 4’,6-diamidino-2-phenylindole (DAPI) and propidium iodide which are highly genotoxic, due to exactly its ability to bind into DNA and damage it, carrying itself a risk for cancer development. We have opted by Giemsa stain even knowing it is a less specific stain and has been criticized due its ability to stain the keratin bodies, usually present into of epithelial cells, which may be misinterpreted as MN and lead to false-positive results (25). The experiment, in fact, demonstrate that the keratin bodies within the buccal epithelial cells may difficult the scoring, however, keratin bodies may be differentiate from MN strictly following the scoring criteria, nevertheless, it is a time consuming procedure and to our experience it took 3 full hours for a single slide check. This technique on the other hand, showed to be feasible without loss compared to the conventional technique which apply a considerable larger number of steps and chemical reagents.

Aware of the difficulties and based on the great variability of techniques, methods and criteria, the “Human MicroNucleus (HUMN) collaborative programme” tried to unify the protocol for MN essay (6,8,26,27), however, advantages as costs and method simplicity were lost, while there was a gain in specificity and slides reading speed. Besides the effort to unify the MN technique, Mahimkar *et al.* (2010) (28) confirmed that the FMic frequencies in Giemsa-stained slides are correlated with genetic polymorphisms corroborating that the applied stain method in our study is valid.

In a review study Bracesco *et al.* (2001) (29) showed that the evidence implicating IP heavy consumption with some neoplasias are inconclusive and on the other hand, those authors declared that several studies confirm the antimutagenic effects of IP in different models. Related to oral and oro-pharyngeal cancer, another systematic review and meta-analysis study (30) observed that odds ratio for Mate tea drinkers was 2.11 (95% CI = 1.39 – 3.11) and the Population Attributable Risk of 16%. Considering the experimental data concerning the genetic toxicity of IP are obscure and contradictory, Wnuk *et al.* (2009) (31) re-evaluated the effects of mate on cell culture of human peripheral lymphocytes and observed that IP increased the FMic by 3.3 fold at a concentration of 10µg/ml and suggest that IP extract may have cytotoxic and genotoxic activity.

Within the limitations of this study and the period of exposure we could observe that mate tea did not induce an increase of FMic in exfoliated buccal cells of healthy nondrinkers and nonsmokers subjects and may not have genotoxic effect, however, like others authors already suggest, more human studies are needed before a conclusion can be made on the oral carcinogenic risk of mate tea to humans.
